# Endometriosis, a disease of the macrophage

**DOI:** 10.3389/fimmu.2013.00009

**Published:** 2013-01-28

**Authors:** Annalisa Capobianco, Patrizia Rovere-Querini

**Affiliations:** Division of Regenerative Medicine, Stem Cells and Gene Therapy, San Raffaele Scientific InstituteMilan, Italy

**Keywords:** endometriosis, alternatively activated macrophages, angiogenesis, Tie-2 expressing macrophages, iron, phagocytosis, hypoxia

## Abstract

Endometriosis, a common cause of pelvic pain and female infertility, depends on the growth of vascularized endometrial tissue at ectopic sites. Endometrial fragments reach the peritoneal cavity during the fertile years: local cues decide whether they yield endometriotic lesions. Macrophages are recruited at sites of hypoxia and tissue stress, where they clear cell debris and heme-iron and generate pro-life and pro-angiogenesis signals. Macrophages are abundant in endometriotic lesions, where are recruited and undergo alternative activation. In rodents macrophages are required for lesions to establish and to grow; bone marrow-derived Tie-2 expressing macrophages specifically contribute to lesions neovasculature, possibly because they concur to the recruitment of circulating endothelial progenitors, and sustain their survival and the integrity of the vessel wall. Macrophages sense cues (hypoxia, cell death, iron overload) in the lesions and react delivering signals to restore the local homeostasis: their action represents a necessary, non-redundant step in the natural history of the disease. Endometriosis may be due to a misperception of macrophages about ectopic endometrial tissue. They perceive it as a wound, they activate programs leading to ectopic cell survival and tissue vascularization. Clearing this misperception is a critical area for the development of novel medical treatments of endometriosis, an urgent and unmet medical need.

## INTRODUCTION

Endometriosis is a common condition, affecting a rather large fraction of menstruating women, characterized by the hormone-dependent persistence and growth of vascularized endometrial tissue at ectopic sites, typically the pelvis, with pain and reduced fertility (Lebovic et al., 2001; Giudice and Kao, 2004; Berkley et al., 2005; Bulun, 2009; Luisi et al., 2009; Giudice, 2010; Simoens et al., 2012). Lesions are thought to originate from endometrial fragments shed during menstruations, which reach via the Fallopian tubes the peritoneal cavity. The event, which is likely to physiologically occur in most women, endometriotic and non-endometriotic, is referred to as “retrograde menstruation” (Sampson, 1927). Supporting evidence is mostly indirect. Endometriosis for example only takes place in species that menstruate, such as primates: this suggests that menstruation is required for the disease (D’Hooghe and Debrock, 2002). Shed endometrium is thought to initially adhere to endoabdominal structures, in particular the peritoneal wall and ovaries. Adhesion probably occurs quite commonly in healthy, non-endometriotic women. In * in vitro models* , adhesion requires a relatively short time and is apparently actively supported by mesothelial cells (Lucidi et al., 2005; Nair et al., 2008), with an apparently healthy peritoneum that thus play an important facilitatory role in the further development of endometriotic lesions (Fassbender et al., 2011)**.**

A further necessary step for lesions establishment is the capacity of ectopic endometrial cells to invade the underlying basement membrane. The molecular bases of this phenomenon are partially elucidated. Endometrial tissue has invades even intact serosal membranes, indicating that a previously disrupted peritoneum is not a requirement (Nair et al., 2008).

Invasion in a prerequisite for the organization of the ectopic endometrial cells in tridimensional cysts but is not sufficient: novel vessels are also necessary. Novel vessels originate through sprouting from the adjacent vasculature or incorporation of circulating precursor endothelial cells at sites of vascularization (Becker et al., 2011; Laschke et al., 2011a,b). They ensure the transport of oxygen and nutrients, the excretion of catabolites and the maintenance of fluid balance.

All together the persistence of ectopic endometriotic tissue is associated to uncontrolled growth, invasion of adjacent tissues, defective apoptosis, neoangiogenesis, and sustained local inflammatory responses. These features are not tissue-autonomous, but depend on the peculiar features of the innate immune response to the auto-transplantation of a hormone-regulated tissue at novel sites. Of importance, several differences exist between the eutopic endometrium of women with and without endometriosis including invasive properties and resistance to apoptosis, as well as between ectopic and autologous eutopic endometrium of endometriotic patients (Novembri et al., 2011). This suggests that the inflammatory peritoneal environment influences the behavior of endometrial ectopic cells.

Macrophages in particular are master regulators of the innate response to injured, infected, and neoplastic tissues. As such they are in charge of selecting the appropriate response to restore homeostasis: they include on one hand the identification and destruction of pathogens, infected or transformed cells; on the other hand the activation, proliferation, and differentiation of precursor/stem cell and the generation of neovessel. The perception of the role of macrophages in endometriosis has grown in the last years: in this review we will discuss the most recent data in the literature that name macrophages as defendants, with the charge of contributing to cause human endometriosis.

## METHODS

Literature searches were performed in PubMed, Scopus, and ISI Web of Knowledge databases for publications focusing on the state and functions of macrophages in human and murine endometriosis. The searches included the key words “endometriosis,” “ectopic endometrial lesions,” and “endometrium” were matched with the key words “macrophages,” “Tie-2 expressing macrophages,” “angiogenesis,” “hypoxia,” “innate immunity,” and “ovarian cancer.” The bibliographic research encompasses studies on rodents, non-human primates and humans. No limits were set for publication dates.

### ENDOMETRIUM AND ANGIOGENESIS

Organ vasculature comprises an integrated system of arteries, arterioles, capillaries, venules, and veins that ensures blood circulation and the transport of oxygen and other gases to target tissues. Blood vessels that form during growth and development generally arise by the sprouting from pre-existing vessels, via a process referred to as angiogenesis (Potente et al., 2011). Angiogenesis seldom occurs in the adult organism in normal tissues under physiologic conditions.

Endometrium represents an exception: the tightly regulated variation of ovarian steroids estrogen and progesterone concentrations cyclically triggers the remodeling of the organ vasculature, with angiogenesis and lymphangiogenesis (Girling and Rogers, 2009). Estrogens during the proliferative stage of the menstrual cycle cause the rapid growth of the vasculature. Progesterone during the secretory phase controls the maturation of the capillary sub-epithelial plexus and the development/coiling of spiral arterioles. Finally, the swift hormone withdrawal triggers the endometrial repair in the perimenstrual period.

The molecular mechanisms involved in hormone-regulated remodeling of eutopic endometrial vessels are being actively investigated. The vascular endothelial growth factor (VEGF) family and associated receptors and the angiopoietin–tyrosine kinase with Ig and epidermal growth factor homology domain (angiopoietin/Tie-2) system play an important role, since they connect hormonal levels to vessel remodeling (Girling and Rogers, 2009; Mints et al., 2010; Elsheikh et al., 2011; Lash et al., 2012).

Peritoneal macrophages are a well-characterized source of VEGF and ovarian steroids regulate the production of the growth factor (McLaren et al., 1996). Estrogens act on various macrophage signaling pathways, influencing in particular those related to the ability to sustain the recruitment of inflammatory cells and the remodeling of inflamed tissues, such as mitogen-activated protein kinase (MAPK), phosphatidylinositide-3-kinase/protein kinase B (PI3K/AKT), and nuclear factor-kappa B (NF-κB): as a consequence, a deregulated response to steroids might influence the survival of ectopic endometrial cells and promote the vascularization of the lesions (Cakmak et al., 2009; Pellegrini et al., 2012).

Premenstrual progesterone withdrawal possibly represents the crucial event: during the late secretory phase the demise of the corpus luteum results in the abrupt fall of bioactive progesterone. This triggers an acute inflammatory response with destruction of the upper “functional” layers of the human endometrium (for a thorough and well-written review, see Maybin et al., 2011a): endometrial prostaglandin (PG), PGE2 and PGF2α, are synthesized that act as potent vasoconstrictor on the spiral arterioles. PG production, spiral arteriole vasoconstriction, and local hypoxia in turn regulate the production of chemokines, such as IL-8 (CXCL8) and CXC chemokine ligand 12 (CXCL12) stromal cell derived factor (SDF-1).

Inflammation and chemokine production results in the attraction of neutrophils, that actively contribute to the destruction of the functional layer of the endometrium (Kaitu’u-Lino et al., 2007): typically tissue breakdown is associated with loss of integrity of endometrial vessel and blood cells extravasation; endometrial fragments and blood are then flushed from the uterus to the vagina with overt menstruation.

The inflammatory response has a double-edged action: besides triggering the regulated destruction of the tissue, it initiates and guides the endometrial repair.

Progesterone withdrawal indeed causes a transient constriction of spiral arteries in the top layers of the endometrium, which is supposed to cause hypoxia (Fan et al., 2008). Hypoxia is the stimulus that triggers vessel remodeling in injured and regenerating tissues as well as in tumors. It elicits an adaptive response, which is largely mediated by the hypoxia-inducible transcription factor-1alpha (HIF1α): under hypoxic conditions HIF1α translocates to the nucleus where it enhances and accelerates the transcription of genes with appropriate response elements, including angiopoietin 2 (Ang-2) CXCL12 and VEGF (Wu et al., 2007, 20011; Liu et al., 2011a; Maybin et al., 2011b,c; Wong et al., 2011; Henriet et al., 2012; Sarkar et al., 2012). Other signals induced by hypoxia and PG in epithelial cells and macrophages, such as connective tissue growth factor, have been convincingly suggested to play a role in endometrial repair (Maybin et al., 2012).

Events downstream HIF1α nuclear translocation are not apparently relevant for tissue destruction (Maybin et al., 2011a): in contrast they are required for attraction of macrophages and for their regenerative action in the hypoxic tissue (Du et al., 2008; Wong et al., 2011; Sarkar et al., 2012). For example ablation of HIF1α in tumor models is associated with decreased CXCL12/SDF1α levels and a less effective recruitment of bone marrow-derived macrophages in the tumors (Du et al., 2008).

Of critical importance for the environmental changes associated to endometriosis (see below), the vasculature of ectopic lesions remains dependent on the systemic concentration of hormones. As such, these structures undergo a cyclic remodeling, with synchronized tissue destruction and bleeding. This has been well-established since the pioneering work of Markee (1978), who has described that endometrial fragments transplanted in the eye anterior chamber of rhesus monkeys implant connect to the iris vasculature and grow. However, the tissue abruptly regresses after hormone withdrawal: cyclic destruction of the ectopic tissue is associated to coiling of spiral arterioles, vasoconstriction episodes, and bleeding.

The insight on the physiological control of eutopic endometrium is likely to be crucial to understand why endometriosis occurs. During menstruation red blood cells, hemoglobin, and leucocytes accumulate in the peritoneal fluid of most healthy women (Bokor et al., 2009): this indicates that retrograde menstruation is a fairly common event. Interestingly, retrograde menstruation appears not to be * per se* more frequent or abundant in endometriotic women (Bokor et al., 2009): therefore, the presence of endometrial tissue in the peritoneal cavity is not sufficient to cause endometriosis (Bulun, 2009). The role of the genetic background and/or of environmental factors deserves better attention and will be a hot topic for researchers in the next years.

### MACROPHAGES ARE VERSATILE CELLS

Macrophages have been originally identified as an integral component of the mononuclear phagocyte system (MPS). They derive from bone marrow progenitors that enter the bloodstream as monocytes. Within a relatively short time they reach peripheral tissues where they yield resident macrophages or antigen-presenting cells, including dendritic cells (DCs): the MPS contributes both to pathogen elimination and to housekeeping functions (van Furth and Cohn, 1968; Murray and Wynn, 2011). Macrophages are professional phagocytes, since they are endowed with the molecular machinery necessary to internalize and dispose of extracellular particulate substrates, including microbes or endogenous constituents, such as apoptotic cells or senescent erythrocytes.

Dedicated pattern-recognition receptors (PRRs) are non-clonally expressed by innate immune cells and by macrophages in particular (Palm and Medzhitov, 2009). PRRs have been initially thought to have undergone evolutionary selection because of their ability to selectively identify molecular structures referred to as pathogen-associated molecular patterns (PAMPs) expressed by large classes of microbes (Janeway, 1992). After PAMP recognition, activation of PRRs recruits tightly coordinated events, including: (i) the production of various cytokines that attract and activate leukocytes (Nathan, 2002), arming them for neutralization/elimination of invading pathogen; (ii) the activation of an acute phase response, with the production of conserved soluble PRRs, such as pentraxins (Manfredi et al., 2008). Acute phase proteins in turn tune leukocyte activation and quench their ability to collaterally damage the tissue.

Besides microbial PAMPs, endogenous moieties trigger the PRR activation: damage-associated molecular pattern (DAMP) comprise an array of heterogeneous molecules that are released during cell and tissue necrosis and that via PRR activation elicit inflammation and prompt tissue regeneration even in the context of sterile injuries (Maroso et al., 1996; Bianchi, 2007; Lotze et al., 2007; Rubartelli and Lotze, 2007; Urbonaviciute et al., 2008; Bianchi and Manfredi, 2009; Manfredi and Rovere-Querini, 2010; Zhang et al., 2010a; Castiglioni et al., 2011; Liu et al., 2011b).

Pattern-recognition receptors represent a crucial asset for the phagocytic ability of macrophages: they on one hand interact with the phagocytic substrate and on the other hand activate specific signaling cascades within the phagocyte. The characteristics of the signaling events are crucial to determine which array of soluble signals is produced.

An example is given by the signal transduction associated with the best-characterized family of PRRs, the Toll-like receptors (TLR): TLR 3, 7, and 9 are preferentially expressed in endosomes, where they have access to viral constituents. As a consequence, they activate PI3K/mTOR/S6K with downstream production of antiviral and pro-inflammatory cytokines, such as type 1 interferons: the resulting response eventually leads to virus elimination.

TLR4, which is expressed at the plasma membrane, together with other extracellular moieties such as MD-2 and CD14, recognizes gram-negative bacteria lipopolysaccharide. Its recruitment results, * via* MyD88-dependent and -independent pathways, in NF-kB nuclear translocation and in the recruitment of the kinase pathways with an eventual production of inflammatory cytokines such as tumor necrosis factor (TNF)-α and interleukin (IL)-6: the response is crucial for the control of the infection (Kawai and Akira, 2011). The activation of other PRRs, such as those that recognize dying cells phagocytic tags, prompts the production of immunosuppressive cytokines, such as IL-10 and transforming growth factor β (TGF-β) and of factors implicated in the tissue regeneration and repair (Huynh et al., 2002; Ravichandran, 2011).

Thus, PRRs decipher the code associated to an infected or injured tissue, and thus enable macrophages to activate the innate/acquired immune response that is more functional for restoring homeostasis (Brancato and Albina, 2011; Murray and Wynn, 2011). Conversely, macrophages extensively reprogram their functional properties in response to PAMPs and DAMPs (Nau et al., 2002; Martinon et al., 2010; London et al., 2011).

During sterile inflammation, which is by definition driven by DAMPs exclusively, macrophages play crucial and non-redundant actions in matrix remodeling and angiogenesis, which are required for effective tissue healing (Vezzoli et al., 2010; Bosurgi et al., 2011; Castiglioni et al., 2011; Rock et al., 2011; Vezzoli et al., 2011).

**FIGURE 1:**
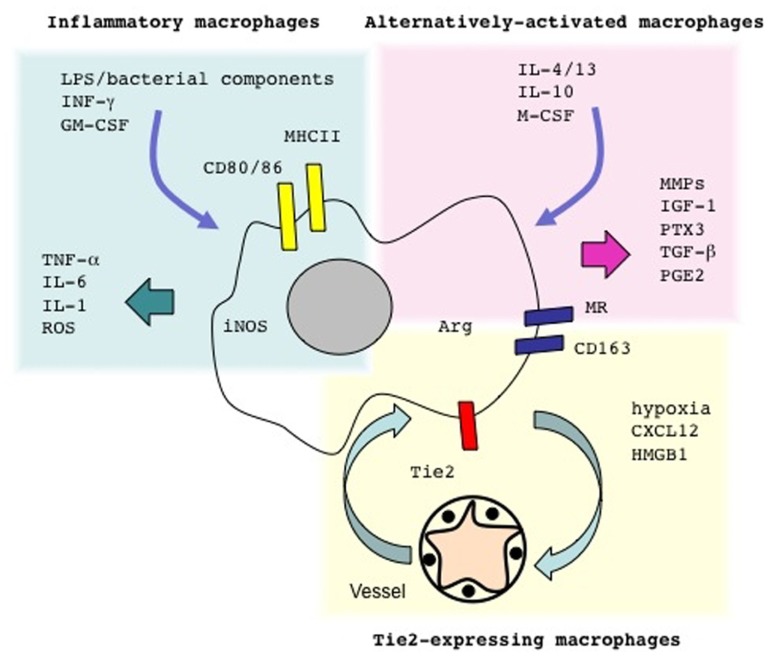
**Macrophages come in different flavors**. Microbial components such as lipopolysaccharide (LPS) and/or cytokines such as IFN-γ or GM-CSF elicit conventionally activated macrophages. These cells secrete inflammatory cytokines and generate molecules involved in the anti-bacterial response, such as reactive oxygen species (ROS). Treatment with different cytokines like IL-4 and/or IL-13, IL-10 and/or M-CSF supports the activation of alternative activated macrophages. Local hypoxia, possibly in conjunction with chemotactic signals such as CXCL12 and/or DAMPs, such as HMGB1, recruit from the blood precursors that yield Tie-expressing macrophages in the inflamed tissues. The expression of specific arrays of membrane receptors and the generation of soluble and gaseous messengers reflect the macrophage activation programs. LPS, lipopolysaccharide; IFN-γ, interferon-γ; GM-CSF, granulocyte macrophage-colony stimulating factor; M-CSF, macrophage-colony stimulating factor; MHCII, major histocompatibility complex class II; CD163, haptoglobin–hemoglobin complex receptor; CD206, mannose receptor; TGF-β, transforming growth factor β; PGE2, prostaglandin E2; ROS, reactive oxygen species.

Macrophages have a rather heterogeneous array of characteristics, which possibly reflects the diverse functions they exert within the microenvironment: they become effector cells that kill invading pathogens, such as mycobacteria or * Leishmania major* and dramatically modify the environment via the production of cytokines and reactive oxygen (ROS) and nitric oxide (NO) species (Darrah et al., 2007; Mylonas et al., 2009). Such macrophages are referred to as classically activated/inflammatory macrophages, or M1 cells (**Figure 1**).

Macrophages also undergo a distinct activation program (“alternative activation”): as a consequence they tune inflammatory responses and adaptive immunity, scavenge debris and promote angiogenesis, tissue remodeling, and repair (Mantovani et al., 2004; Gordon and Taylor, 2005). Macrophages within regenerating tissues are often alternatively activated, also termed “M2 cells” (**Figure 1**; Daley et al., 2010; Gordon and Martinez, 2010; O’Brien et al., 2010; Schwartz, 2010; Brancato and Albina, 2011; Cairo et al., 2011; Corna et al., 2010; David and Kroner, 2011; Harel-Adar et al., 2011; Jaeschke, 2011; London et al., 2011; Wang and Harris, 2011)****In contrast, uncontrolled classical activation in general results in defective healing and persistent inflammation (Gordon, 2003; Sindrilaru et al., 2011)****Recent elegant studies suggest that neural stem cells in a model of murine sterile spinal cord injury possibly direct physically interact with endogenous macrophages, an event that modulates expression levels of inflammatory cell transcripts * in vivo* , with a shift from “classically activated” (M1-like) macrophages to cells that facilitating the healing or regeneration of the lesion (Cusimano et al., 2012). This is not an exception due to the environment of the spinal cord: a similar shift occurs in injured skeletal muscles, apparently facilitated by the signals derived from the apoptotic substrates (Arnold et al., 2007).

Recombinant cytokines can be used to activate macrophage precursors. Alternatively activated macrophages can be propagated * in vitro* by exposing monocytes or bone marrow precursors to low concentrations of macrophage-colony stimulating factor (M-CSF) in the presence of IL-4, IL-13, or IL-10 (Gordon, 2003; Mantovani et al., 2004). Exposure to microbial components in the presence of γIFN or to granulocyte macrophage-colony stimulating factor (GM-CSF) elicits classically activated, inflammatory macrophages.

Specific subpopulations of macrophages are preferentially involved in guiding angiogenesis (Qian and Pollard, 2010; De Palma and Naldini, 2011). Macrophages that express the Tie-2 receptor (TEM or Tie-2-expressing monocytes/macrophages) sustain neoangiogenesis in a variety of experimental tumor models (**Figure 1**). Circulating monocytes in normal conditions express limited amounts of Tie-2: however, they substantially up-regulate it after homing to hypoxic tissue, where they yield a subset of perivascular macrophages (Du et al., 2008; De Palma and Naldini, 2009; Squadrito and De Palma, 2011). Angiopoietins, cytokines that regulate the quiescent and the angiogenic microvasculature, are known ligands of Tie-2: * in vivo* studies in which TEMs had been specifically depleted demonstrate that they are required to support angiogenesis and accelerate tumor growth (De Palma et al., 2003, 2005), indicating that TEMs interaction with the angiogenic vasculature plays a non-redundant role.

The model that apparently better fit with the data obtained in these diverse systems regards macrophages as guardians of the tissue integrity, that are in charge of: (i) perceiving clear and actual injury in the tissue; (ii) clearing dying cells and tissue debris; (iii) regulating neovessel generation and extracellular matrix remodeling; (iv) sustaining stem and progenitor cells migration, proliferation, survival, and differentiation (Lolmede et al., 2009; Stappenbeck and Miyoshi, 2009; Sun et al., 2009; Vezzoli et al., 2010; Zhang et al., 2010b; Ehninger and Trumpp, 2011; Hara et al., 2011; London et al., 2011).

Macrophage activation is effective at enforcing regenerative and vascular responses that conduce to tissue repair with restitutio ad integrum when original injuries are intense and short lasting. In contrast, it is likely to be detrimental when the cause(s) of homeostasis disruption cannot be eliminated. We speculate that this applies to human endometriosis.

### ENDOMETRIOSIS, HYPOXIA, AND MACROPHAGES

Angiogenesis is a prerequisite for endometriotic lesions to establish and to grow * in vivo* . Endometriotic lesion neovascularization involves both conventional sprouting angiogenesis and actual vasculogenesis (Laschke et al., 2011a): the process shares several features with the vessel remodeling process that take play in injured tissues and is required for wound healing (Potente et al., 2011). It is physiologically dependent on macrophage activation, since the depletion of macrophages in experimental models of wound healing jeopardizes VEGF generation and disturbs neovascularization (van Amerongen et al., 2007; Martinez et al., 2008). Reduced/delayed differentiation of myofibroblasts, re-epithelialization, collagen deposition, and cell proliferation also ensue macrophage depletion and neovessel disruption (Mirza et al., 2009).

A large body of evidence indicates that macrophages are responsible for the angiogenic switch, i.e., the increase in the density of vessels that often characterizes the benign-to-malignant transition in cancer, being involved both in the initial establishment of the vasculature and in the subsequent remodeling of the vessels (Pollard, 2008). Their action is mediated via several pathways, that lead to: (i) the production of matrix metalloproteinases (MMPs) that are required for the release of VEGF bound to the extracellular matrix at hypoxic sites, thus increasing the availability of the bioactive growth factor; and (ii) the expression of VEGF family members, which in turn behave as attractors of myeloid cells, including macrophages. Of interest, an association between endometriosis and VGEF-A gene polymorphisms has been reported, even if caution should be used because of the effect of possible confounding factors.

In experimental models of endometriosis, early phases of lesion establishment are characterized by a transient hypoxia, which results in the up-regulation of HIF1α, with downstream expression of VEGF (Wu et al., 2007, 20011; Becker et al., 2008; Lin et al., 2012). Limited ischemia of the endometrium in the early and middle secretory phase occurs: the event is apparently associated with the up-regulated expression of VEGF in the late secretory phase of the menstrual cycle in endometriotic women (Donnez et al., 1998). Interestingly, endometrial fragments from women in which a transient ischemia had been induced by repeated clamping/declamping of the uterine artery transplanted onto the chick embryo chorioallantoic membrane demonstrated higher VEGF expression and better survival: this mechanism could facilitate implantation/establishment of endometrium at ectopic sites (Ren et al., 2011). Moreover, response to ischemia is likely to play a role in established lesions of endometriotic patients: the relative expression of HIF1α and VEGF differ at various sites within endometriotic lesions, possibly accounting for some of their heterogeneous histological characteristics (Goteri et al., 2004, 2010).

It is important to underline that the vascular response to ischemia depends on the recruitment of activated macrophages at lesions sites (e.g., see Machado et al., 2010). Macrophages are indeed necessary to license lesions for growth and spreading (Bacci et al., 2009; Haber et al., 2009; Capobianco et al., 2011 and see below).

### MACROPHAGES AS A KEY TO THE SUSCEPTIBILITY TO ENDOMETRIOSIS

**FIGURE 2:**
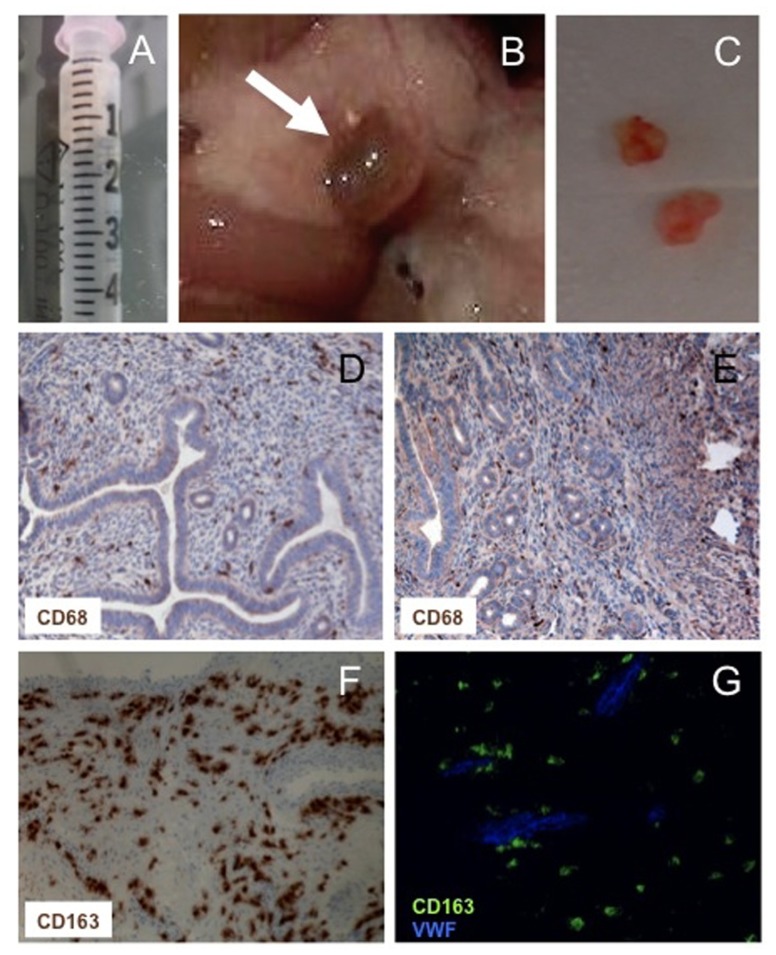
**Macrophages in experimental and human endometriosis**. Endometrial fragments (maximal diameter <1 mm) derived from estradiol benzoate-treated mice **(A)** injected intra-peritoneally yield endometriotic lesions **(B)** that can be excised and processed for disease assessment **(C)** or immunohistochemical evaluation **(D–G)**. CD68^+^ macrophages are abundant in human and experimental lesions **(D,E)**. Most macrophages within human lesions express markers of alternative activation, such as the hemoglobin/haptoglobin CD163 scavenger receptor **(F)**. CD163^+^ (green) vWF- (blue) pro-angiogenic Tie2-expressing macrophages surround the neoformed vessels **(G)**.

**FIGURE 3:**
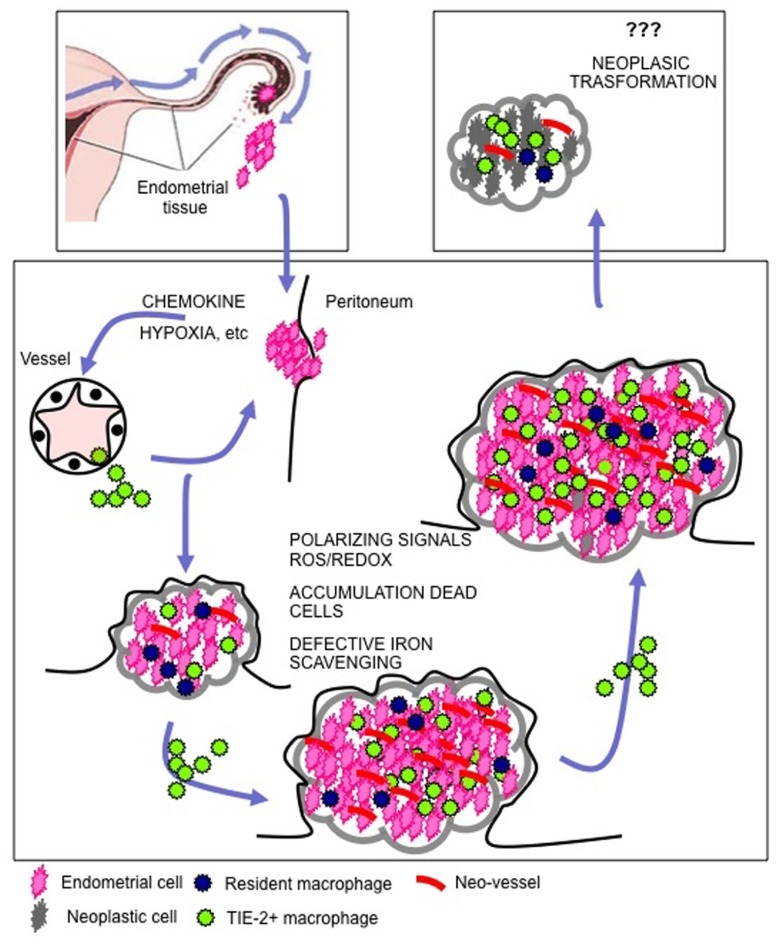
** Macrophages drive endometriosis**. Endometrial cells reach the peritoneal cavity as a consequence of retrograde menstruation. The recruitment of macrophages at the site of endometrial cell engraftment is a limiting step for the lesion to organize and to growth. Local hypoxia and the production of chemokines play a major role in the recruitment of macrophages. Once endometriosis is established, the cyclic death of endometrial cells as a consequence of progesterone withdrawal leads to the release of cell debris, erythrocytes, and heme-bound iron in the peritoneal cavity. Recruited macrophages perceive ongoing cell death and tissue damage: in endometriotic patients they activate a reparative/regenerative/angiogenic program that is required for lesion maintenance, growth and spreading. In a subgroup of patients endometriosis is a preneoplastic condition. The persisting tissue healing action of macrophages that keep interfering with the physiological apoptosis while prompting proliferation of epithelial cells might set the scenario in which genetic alterations accumulate.

The data discussed above indicate that endometriosis depends on the ability of endometrial tissue at ectopic sites to attract macrophages and to be as a consequence licensed for survival, angiogenesis, growth, and spreading (**Figures 2 and 3**). Macrophages physiologically convey these licensing signals in the eutopic endometrium, which undergoes physiological destruction as a consequence of progesterone withdrawal**.**

Infiltrating macrophages are a consistent feature of endometriotic lesions. Independent lines of evidence indicate that they undergo activation as a consequence of signals generated within ectopic lesion (Lebovic et al., 2001; Zhang et al., 2006; Herrmann Lavoie et al., 2007; Lawson et al., 2007; Minici et al., 2007; Galleri et al., 2008; Lousse et al., 2008) or possibly of the lack of hormone-regulated anti-inflammatory signals in the ectopic but none in the eutopic endometrium (Novembri et al., 2011). However, it is unclear whether these changes in eutopic endometrium are primary defects or consequences of the development of endometriosis (Novembri et al., 2011). Ectopic endometriotic lesions are histologically similar to their putative eutopic precursors. However, several genes are differentially expressed in ectopic and eutopic of endometriotic and non-endometriotic patients and significant biochemical differences exist (Meola et al., 2010). These differences might stem from epigenetic modifications that occur in the eutopic endometrium of patients with endometriosis. Differential methylation and expression of genes involved in the regulation of implantation, such as * HOXA10* and * PR-B, * have been for example reported in the eutopic endometrium of patients with endometriosis compared with controls (Stilley et al., 2012).

In endometriotic lesions the macrophage NF-kB-dependent pathway is engaged (Lousse et al., 2008) with transactivation of responsive gene elements that control angiogenesis and tissue remodeling (Hagemann et al., 2008; Timmer and Nizet, 2008). NF-kB inhibition decreases the * in vivo* growth of experimental lesions, indicating that its activation plays a particularly relevant role (Lousse et al., 2008, 2009).

The stimuli involved in the activation of macrophages are so far unclear. It is extremely unlikely that microbial structures (PAMPs) are involved. As such, the signals involved in the macrophages activation must be released in sterile conditions, like it happens for multiorgan trauma, which causes the systemic inflammatory response syndrome or at specific sites, as a consequence of ischemia/reperfusion or of immune mediated events, like in the case of allograft rejection. The endogenous sterile triggers that are responsible for sterile inflammation in these conditions are apparently equally effective as microbial PAMPs and rely on similar molecular pathways; NF-kB nuclear translocation and activation is a clear example (Palumbo et al., 2007).

Once ectopic lesions are established, tissue remnants that are generated as a consequence of cyclic response to progesterone withdrawal accumulate in the peritoneal cavity since they cannot be flushed out with the menstrual blood. Cell remnants have a potentially noxious action on living tissues: as such, they require to be disposed by active phagocytosis. This is in general a relatively silent event, with macrophages recognizing and phagocytosing scattered cells that die in the midst of living tissues because of their physiological tissue turnover, releasing in the meantime immunosuppressive and anti-inflammatory signals. However, the challenge with a relatively ample load of cells dying in a synchronized fashion may exert a paradoxically opposite effect, representing a further threat to tissue integrity and immune tolerance (Rovere et al., 1998, 2000; Manfredi et al., 2002; Capobianco et al., 2011). Specifically the release of intracellular moieties endowed with inflammatory and adjuvant properties results in the activation of macrophages, which in turn release cytokines and chemokines, thus enforcing a positive feed-back forward loop (Zhang et al., 2010a; Castiglioni et al., 2011). The persistence of cells dying as results of progesterone withdrawal within endometriotic lesions could cyclically activate infiltrating macrophages, thus sustaining the inflammation associated to the disease.

Relatively little is known on the ability of peritoneal macrophages to dispose of endometrial remnants, even if an apparent impairment in the ability to phagocytose particulate substrate has been reported (Chuang et al., 2009). The defect has been associated with a defect in the expression and the function of the class B scavenger receptor CD36, a well recognized player in the recognition and clearance of apoptotic cells, in endometriosis: such defect could be possibly related to the action of PG (Chuang et al., 2010). However, it is difficult to verify whether such defect is upstream or downstream the persistent inflammation of the peritoneal cavity associated to the disease.

Ectopic endometrium breakdown causes hemorrhage, with extravasation and persistence of aging red blood cells within the lesions; moreover erythrocytes accumulate into the pelvis because of retrograde menstruation (Defrere et al., 2008, 2012; Lousse et al., 2009, 2012; Capobianco et al., 2010). The transfer of human menstrual effluent in immunodeficient mice results in the generation of iron deposits, which are similar to those observed in patients (Defrere et al., 2006), demonstrating that the specific features of the peritoneal microenvironment do not allow an effective processing of red blood cells, in contrast to other professional hemocatheretic tissues, such as the spleen.

The clearance of red blood cells and of apoptotic remnants has similar molecular constrains and often relies on common phagocytic tags/receptors pairs, which are in general expressed by professional phagocytes, such as macrophages (Ravichandran, 2011). The peritoneal fluid of endometriotic women indeed contains iron-loaded macrophages and higher fluid concentrations of the metal, which are possibly associated with the severity of the disease manifestations (Lousse et al., 2012). The iron in the fluid possibly derives from the lysis of red blood cells that that are not effectively cleared by macrophages, and as a consequence release hemoglobin. This moiety may as well bind to haptoglobin and be internalized by macrophages via the CD163 hemoglobin/haptoglobin scavenger receptor. In endometriosis patients this pathway is either originally defective or peritoneal macrophages become insufficient/unable to deal with the amounts of hemoglobin present in the liquid. The concentration of free iron within endometriotic cysts has also been investigated and found to be high. Metal accumulation was reflected by a higher oxidative stress, which in turn favors epigenetic modifications, prompting the evolution of ectopic tissue in * bona fide* neoplastic lesions (Yamaguchi et al., 2008, 2010).

Macrophages are also able to directly recognize senescent erythrocytes, internalize and digest them, an event which in the bone marrow is necessary for the active recycling of heme-iron for effective erythropoiesis (Li and Ginzburg, 2010; Ganz, 2012). Peritoneal macrophages are known to accumulate iron (Lousse et al., 2009). So far relatively little information is available on the characteristics of the macrophages disposal of red blood cells and heme-iron within endometriotic lesions, in particular when progesterone withdrawal jeopardizes the integrity of the vessel walls.

All together iron homeostasis is disrupted in the peritoneal cavity of patients with endometriosis: the accumulation of unscavenged iron associates with the increased generation of ROS and with a persistently activated pathway of the NF-kB: alterations of this pathway have been reported in eutopic endometrium of patients with endometriosis, suggesting a possible causative role in the natural history of the disease (Ponce et al., 2009). Interestingly polymorphism of the NFKB1 promoter have been described that are significantly associated with an increased risk to develop endometriosis (Zhou et al., 2010).

The peritoneal environment, whose characteristics are modulated by a vast and complex array of regulatory peptides and growth factors (Petraglia et al., 2008; Florio et al., 2009), could further promote and amplify the function of alternatively activated macrophages. Immune and non-immune cells for example produce urocortin neuropeptides, especially under inflammatory stimuli and hypoxic conditions (Buhler et al., 2009; Imperatore et al., 2010). Neuropeptides have been involved in the resolution/termination of the innate response, possibly via regulation of the production of inflammatory cytokines and of endothelial permeability (Di Comite et al., 2007, 2009). It has been recently shown the various members of the urocortin families are differentially expressed in ectopic and eutopic endometrium: their expression is finely regulated throughout the menstrual cycle in the eutopic tissue, while virtually no menstrual-cycle-related changes were found in endometriotic lesions (Novembri et al., 2011). Intriguingly, urocortin down-regulates secretion by activated macrophages of the best-characterized DAMP signal, the high-mobility group box 1 (HMGB1) nuclear protein, suggesting that macrophages are major targets in its inhibitory activity (Chorny and Delgado, 2008). HMGB1 constitutively promotes angiogenesis and is used by inflammatory macrophages to attract vessel-associated stem cells and to favor tissue remodeling (Campana et al., 2009; Lolmede et al., 2009). HMGB1 physically associates with CXCL12 and specifically modulates the CXCL12/CXC chemokine receptor 4 (CXCR4) pathway (Zhao et al., 2007; Campana et al., 2009; Schiraldi et al., 2012), i.e., a crucial chemotactic pathway involved in the recruitment of phagocytes and of stem cells at sites of hypoxia. Other endogenous negative signals regulating HMGB1 action have been described (Bianchi and Manfredi, 2009), but their involvement in the pathogenesis of endometriosis have not so far been extensively studied.

### THE DEVELOPMENT OF ENDOMETRIOSIS DEPENDS ON MACROPHAGES

Animal models represent a useful tool to study * in vivo* early steps of the natural history of endometriosis, which would be impossible to tackle in patients. Small animals and rodents in particular have several advantages and have prompted in the last decades a substantial in our insight of the pathogenesis of the disease: they are relatively inexpensive, inbred animals are available, including genetically modified models, in which the endometrial tissue can be transferred and relevant biological events evaluated in reference to the characteristics of the developing lesions (Becker et al., 2006; Tirado-Gonzalez et al., 2012). Lesions in murine models are surgically induced, for example by micro-laparatomic techniques (Nisolle et al., 2000) or induced by injection of endometrial tissue of various origin within the peritoneal cavity (Somigliana et al., 1999, 2001; Fainaru et al., 2008). The latter approach, as originally described, rely on the transfer of fragments of endometrial tissue harvested from syngeneic donor mice and recapitulates important aspects of the disease, comprising hormone-dependence, ability to escape immune surveillance, and characteristics of the neovascularization of ectopic endometrium (Somigliana et al., 1999). Endometriotic lesions are known to originate only in some mouse strains, a feature that resembles the heterogeneous sensitivity to the diseases of the human population.

It must however be underlined that these models have substantial disadvantages (Tirado-Gonzalez et al., 2010): in particular, rodents do not menstruate and thus do not * per se* develop endometriosis. As a consequence biological events are missing that are substantially influence the natural history of the diseases, such as the cyclic disruption of the ectopic endometrium as a consequence of variation of the systemic concentration of sexual hormones (discussed above) and the accumulation of iron in the lesions and the peritoneal cavity.

We have used an experimental mouse model (**Figure 2**) to verify whether macrophages are actually required and influence the establishment of endometriotic lesions. We have depleted macrophages by the intraperitoneal injection of clodronate encapsulated into liposomes: this system results in the selective targeting of clodronate to macrophages, that are killed without undue toxicity on cells belonging to other lineages (Van Rooijen and Sanders, 1994; Bacci et al., 2009; Cottone et al., 2011). We have observed that in the absence of macrophages, syngeneic endometrium retains the ability to adhere to the peritoneal layer and to infiltrate the serosal membrane. However, ectopic lesions fail to grow in these conditions. The pharmacological depletion of macrophages at later times, when endometriotic lesions have already established and organized, “congeal” them: vessels do not extend to the lesions core, which stop growing and do not develop a well-organized glandular and stromal architecture (Bacci et al., 2009).

The data suggest that the recruitment of macrophages into the lesions is not only an early event in the lesion development, but a necessary step for the successful establishment of endometriotic lesions. Interestingly, a similar differential sensitivity of the early phases of the diseases, which are not affected, versus the later growth neovascularization and spreading of lesions characterizes immunodeficient mice that do not express the Tgfb1 transplanted with human eutopic endometrial tissue (Hull et al., 2012). Members of the TGF-β family have been implicated both in the initiation of menstruation and in repair of eutopic endometrium (Omwandho et al., 2010). Moreover, TGF-β is critically expressed in endometriotic lesions (Omwandho et al., 2010).

Transforming growth factor β locally increases the expression of the PAR2 gene with possible downstream increased secretion of IL-6 from endometriotic stromal cells thus suggesting an upstream role of the cytokine in coordinating the cascade of inflammatory signals of the disease (Saito et al., 2011). TGF-β is a key factor produced by macrophages challenged with apoptotic cell remnants (Xiao et al., 2008), which as such could represent a relevant source of the cytokine.

Macrophage dependence is not limited to mice, since initiation and growth of endometriotic lesions are both jeopardized in a rat model of the disease after depletion of peritoneal macrophages by local injection of liposomal alendronate (Haber et al., 2009). Of interest, in this model the degree of lesion infiltration by macrophages is directly correlated with the peritoneal concentrations of TNF-α, while those of the CC chemokine ligand 2 (CCL2)/monocyte chemoattractant protein 1 (MCP-1) chemokine are negatively correlated with the degree of macrophage infiltration (Haber et al., 2009). As discussed above, macrophages deliver trophic and anti-apoptotic signals. These effects in the early phase after endometrium injection are likely to be more relevant (Lin et al., 2006), before novel vessels have established (Eggermont et al., 2005; Grummer, 2006; Becker et al., 2008), which promote the survival of ectopic cells in an hypoxic environment (Lin et al., 2006).

### REPARATIVE AND PRO-ANGIOGENETIC MACROPHAGES IN ENDOMETRIOSIS

Macrophages from patients with endometriosis and mice with implanted endometriotic lesions (but not peritoneal macrophages from non-endometriotic patients or from control mice) express typical markers of alternative activation, in particular high levels of scavenger receptors, CD163 and CD206 (Bacci et al., 2009). CD206 belongs to the C-type lectin superfamily is a well-characterized PRR and contributes to remove or inactivate inflammatory signals (Allavena et al., 2004). CD163 mediates endocytosis of haptoglobin-hemoglobin complexes, with degradation of heme-iron components that can be recycled for erythropoiesis (Kristiansen et al., 2001; Borda et al., 2008), a feature that may be particularly important in a disease in which disturbance of macrophage iron homeostasis appear particularly important (see above). Macrophage polarization results in differential iron management in both human and mice, with classically activated M1 macrophages that are characterized by iron sequestration, which operates as a bacteriostatic mechanism (Cairo et al., 2011).

In contrast alternatively activated M2 macrophages are endowed with the ability to effectively internalize and recycle the metal, with is reflected by a larger intracellular labile iron pool (Corna et al., 2010; Recalcati et al., 2010). The ability to uptake and recycle the metal to bystander cells could be relevant for the repair of tissues in which large amount of heme-iron are expressed, such as the skeletal muscle or the myocardium (Brunelli and Rovere-Querini, 2008; Corna et al., 2010) and may conversely be involved in sustaining the proliferation of neoplastic cells and the growth of neoplastic lesions (Recalcati et al., 2010; Cairo et al., 2011). It is tempting to speculate that the skewing of endometriotic macrophages toward alternative activation results in a more effective transfer of the metal to epithelial cells, with the effect to support the growth and the spreading of the lesions. This model would nicely fit with the increased extracellular concentration of iron in the peritoneal fluid of patients with endometriosis, as well as with the relative overload of macrophages with the metal (Defrere et al., 2012).

Macrophages are alternatively activated in the inflammatory peritoneal fluid or in the endometriotic lesions, both in patients and experimental animals (Bacci et al., 2009). This suggests that this program is important for the natural history of the disease. To experimentally address this possibility, we have set up a model of cell transfer with various polarized macrophage populations in mice in which the endogenous macrophage population had been previously depleted (see above). In this system, alternatively activated macrophages strongly enhance the growth of endometriotic lesions. In contrast mice injected with conventionally activated inflammatory macrophages develop minute lesions, that do not grow and are characterized by a severely disrupted glandular and stromal architecture (Bacci et al., 2009).

Given the critical role of lesions neovascularization in their outcome, we have focused on the possible role of TEMs, the best-characterized population of macrophages involved in angiogenesis: this subset has mainly been characterized in tumor models (De Palma and Naldini, 2011) but it has convincingly been suggested to play a role even in non-neoplastic conditions, in particular in the physiological development of embryos (Pucci et al., 2009). We have observed that human TEMs infiltrate areas surrounding endometriotic novel vessels (Capobianco et al., 2011). To obtain a mouse model in which TEMs, and not Tie2-expressing endothelial cells, are targeted we have transplanted in wild-type recipients bone marrow progenitor cells expressing a suicide gene under the Tie2 promoter/enhancer. In this system, TEMs effectively infiltrated endometriotic lesions, whose growth abruptly abates after TEM depletion. Of interest, in the absence of TEMs endothelial cells caspase 3 is activated. This results in disruption of vessel integrity and of glandular architecture, suggesting a paracrine action of signals delivered by TEMs on the survival of endothelial cells within the neovessel wall.

### ENDOMETRIOSIS AS A PRENEOPLASTIC CONDITION: A ROLE OF MACROPHAGES?

Endometriotic tissue comprises non-transformed cells only. However, it shares several features with neoplasms (uncontrolled growth, invasion of adjacent tissues, defective apoptosis, sustained local inflammatory responses). Conversely, endometriosis increases the risk of ovarian cancer, in particular invasive low-grade serous, clear-cell, and endometrioid subtypes of the neoplasm (Yamaguchi et al., 2008, 2010; Pearce et al., 2012). The molecular bases of the association are not completely defined. As discussed above, macrophages are physiologically recruited in injured tissues, where they activate the neo-angiogenic switch, sustain resistance to apoptotic stimuli and stimulate the proliferation and invasion of precursor cells, in order to prompt tissue regeneration. Macrophages recruited in the endometriotic lesions activate a similar program: they interfere with physiological apoptosis while prompt proliferation, thus setting the scenario in which genetic alterations accumulate. This action may contribute to the evolution of lesions toward atypical endometriosis and metaplasia, which in turn fosters the development of borderline and finally fully malignant ovarian cancer (Wei et al., 2011). Further studies are required to experimentally address this possibility.

## CONCLUSION

Endometriosis represents an immunological unique scenario, since it derives from the auto-transplantation of endometrial cells at distant sites. Recent studies have identified lesional macrophages as responsible for the outcome of the auto-transplantation, since their unrestrained activation toward a reparative phenotype allows the survival, the neovascularization, and the growth of the lesions. This event possibly depends on the transfer at distant sites of inflammatory regenerative mechanisms that are physiologically activated to repair the eutopic endometrium during the menstrual cycle. Clarifying the molecular mechanisms of the misperception of macrophages is a critical area for the development of novel medical treatments of endometriosis, an urgent and unmet medical need.

## Conflict of Interest Statement

The authors declare that the research was conducted in the absence of any commercial or financial relationships that could be construed as a potential conflict of interest.
